# The effect of network structure on epileptic dynamics: analysis of the synchronisation properties of an inter-network of cortical columns

**DOI:** 10.1186/1471-2202-12-S1-P46

**Published:** 2011-07-18

**Authors:** Andre DH Peterson, Iven MY Mareels, Anthony N Burkitt, David B Grayden, Hamish Meffin, Mark J Cook

**Affiliations:** 1 St. Vincent’s Hospital Melbourne, Victoria 3065, Australia; 2 Department of Electrical & Electronic Engineering, University of Melbourne, Victoria 3010, Australia; 3 The Bionic Ear Institute, East Melbourne, Victoria 3002, Australia; 4 NICTA Victoria Research Laboratory, Victoria 3010, Australia

## 

Focal epilepsy is characterised by the spread of hyper-synchronous seizure activity from pathological cortical tissue (focus) to other parts of the surrounding cortex [1]. Our research will form the basis of a mathematical description of a mesoscopic network of cortical columns, where the network dynamics of seizure-like behaviour will be examined as it spreads from a focal (pathological) column to other columns. Emphasis is on how the local dynamics and the network topology influence the overall global dynamics of the seizure spread. Most of the brain’s connectivity (white matter) is heterogeneous and anisotropic with only the local connections (within a column) being approximately homogeneous. The majority of mesoscopic neural models do not model any spatially heterogeneous or anisotropic structure within the cortex as they quickly become mathematically intractable [2]. The aim of this study is to examine the dynamics of an inter-network of populations of neurons that approximate a heterogeneous inter-network of cortical columns through the structure of a connectivity matrix as opposed to uniform connectivity. Analysis of the behaviour of this inter-network demonstrates the dependence of the dynamics on both the structure of the connectivity matrix and the neural model used either spiking or neural field.

The mathematical formalism of complex network theory allows us to examine the relationship between the connectivity and dynamics of a network of cortical columns. By understanding this relationship, the structure of the network can be used to constrain the dynamics so that an order-reduction of a more complicated model can be performed on the network making the model significantly more mathematically tractable.

**Figure 1:**
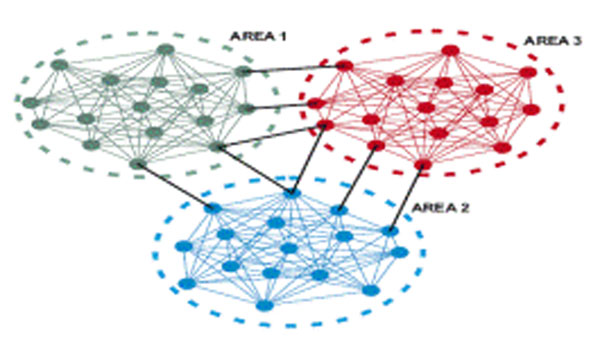
[3]

A network of cortical columns is approximated by modelling each column as an area (see Fig. 1) that has densely connected nodes (intra-population), where each area or column is sparsely connected (inter-population). Singular perturbation methods are used to perform a time-scale separation of the dynamics of the nodes and areas; i.e., the solutions evolve in two different time scales separated by a boundary layer. The time-scale separation can then be used to perform an order reduction of the higher dimensional system into a low-dimensional model that predicts the dynamics of the full model [3]. Epileptic dynamics are examined by analysing the synchronisation of both the intra-population and inter-populations of neurons. The individual nodes synchronise on the fast time scale and these become aggregate nodes on the slow time scale; i.e., the synchronisation within a column compared to the synchronisation between columns. These preliminary results show that the structure of the connectivity matrix has a far greater effect on the dynamics than the type of neural model used, in this case a leaky integrate-and-fire model.

This work is the first stage necessary for constructing a physiologically plausible mathematical model of a mesoscopic network of cortical columns that includes more realistic heterogeneous and anisotropic connectivity. Future research will be directed at incorporating an epileptic focus into the network of columns in order to investigate seizure spread. In particular, the relationship between network topology and dynamics will be examined and how this affects the spread of a seizure.
